# The clinical efficacy of reminiscence therapy in patients with mild-to-moderate Alzheimer disease: Study protocol for a randomized parallel-design controlled trial

**DOI:** 10.1097/MD.0000000000009381

**Published:** 2017-12-22

**Authors:** Mo Li, Ji-hui Lyu, Yi Zhang, Mao-long Gao, Wen-jie Li, Xin Ma

**Affiliations:** a Center for Cognitive Disorders; b Department of Scientific Research and Teaching; c The Geriatric Institute for Clinic and Rehabilitation, Beijing Geriatric Hospital; d Center of the Treatment in Depressive Disorders, Beijing Anding Hospital, Capital Medical University; e Beijing Key Laboratory of Mental Disorders, Department of Psychiatry, Beijing Anding Hospital, Capital Medical University, Beijing, China.

**Keywords:** Alzheimer disease, clinical trial, reminiscence therapy

## Abstract

**Introduction:**

Alzheimer disease (AD) is one of the most common diseases among the older adults. Currently, various nonpharmacological interventions are used for the treatment of AD. Such as reminiscence therapy is being widely used in Western countries. However, it is often used as an empirical application in China; the evidence-based efficacy of reminiscence therapy in AD patients remains to be determined. Therefore, the aim of this research is to assess the effectives of reminiscence therapy for Chinese elderly.

**Methods and analysis:**

This is a randomized parallel-design controlled trial. Mild and moderate AD patients who are in the Beijing Geriatric Hospital, China will be randomized into control and intervention groups (n = 45 for each group). For the intervention group, along with conventional drug therapy, participants will be exposed to a reminiscence therapy of 35 to 45 minutes, 2 times/wk for 12 consecutive weeks. Patients in the control group will undergo conventional drug therapy only. The primary outcome measure will be the differences in Alzheimer disease Assessment Scale-Cognitive Section Score. The secondary outcome measures will be the differences in the Cornell scale for depression in dementia, Neuropsychiatric Inventory score, and Barthel Index scores at baseline, at 4 and 12 weeks of treatment, and 12 weeks after treatment.

**Ethics and dissemination:**

The protocols have been approved by the ethics committee of Beijing Geriatric Hospital of China (approval no. 2015-010). Findings will be disseminated through presentation at scientific conferences and in academic journals.

**Trial registration:**

Chinese Clinical Trial Registry identifier ChiCTR-INR-16009505.

## 1 Introduction

Alzheimer disease (AD) has been recognized as a progressive neurodegenerative disease with the common clinical manifestations of cognitive loss, problems with language, disorientation, loss of motivation, inability to perform self care, and the other neuropsychiatric and behavioral issues.^[1,2]^ Moreover, AD is a major cause of dementia, which is reported to account for approximately 60% to 70% of dementia cases.^[2]^ In developing countries such as China, evidence from epidemiological studies suggested that AD has become the fourth leading cause of death in the elderly, surpassed only by heart disease, cancer, and cerebrovascular disease.^[3]^ Therefore, AD has become a serious cause for the morbidity and mortality of the elderly in both developed and developing countries.

In contrast to the growing burden of AD-related health problems, effective strategies for the prevention and treatment of AD are rare. It has been indicated that patients suffering from AD are vulnerable to abnormalies in behavioral and psychological symptoms, such as depression, irritability, agitation, aggression, and delusions during the overall course of the disease.^[4]^ Accordingly, large numbers of prescriptions for antipsychotic and antidepressant drugs are dispensed to control the behavioral and psychological symptoms in patients with AD. However, the overall benefits of these medications are still under debate, because these antipsychotic and antidepressants drugs have the potential to cause serious side effects, such as metabolic disorders and stroke related mortality.^[5]^ On the contrary, increasing evidence suggests that nonpharmacological treatment for dementia associated with AD and other diseases may be optimal for the relieving of the physical and psychological symptoms, and more importantly, do not have serious adverse effects.^[6]^ Currently, various nonpharmacological treatments are used for the treatment of AD-related dementia, including behavioral intervention, music therapy, multisensory stimulation therapy, and reminiscence therapy,^[7]^ of which, reminiscence therapy is suggested to be of great potential.^[8]^ According to the definition from the American Psychological Association, reminiscence therapy is defined as the use of life histories—written, oral, or both to improve psychological well-being.^[9]^ Since the first performance of reminiscence therapy in 1963 by Butler, reminiscence therapy has now been applied in patients with chronic conditions, depression, and dementia from Western countries, Japan, Taiwan, and other regions of the world.^[10–18]^ Results of some pilot clinical trials have indicated the efficacy of reminiscence therapy for the improvement of cognitive function and overall quality of life, particularly the emotional function and overall happiness in these patients.^[19–24]^ However, in developing countries like China, reminiscence therapy is performed based on the judgment of the physician, and the evidence-based efficacy of reminiscence therapy in these patients remains to be determined. Therefore, we designed this randomized parallel-design controlled trial to evaluate the efficacy of reminiscence therapy on the cognitive, emotional, behavioral and psychological symptoms, as well as daily living ability of patients with AD. Results of our study may provide primary scientific evidence for the using of reminiscence therapy in AD-related dementia in China.

## 2 Main objective

This study aims to assess the effects of reminiscence therapy on cognitive, emotional, behavioral, and psychological symptoms, daily living ability, and other aspects in patients with mild and moderate AD in addition to conventional drug treatment.

## 3 Methods/design

### 3.1 Study design

This randomized parallel-design controlled trial will be conducted in the Beijing Geriatric Hospital of China. A total of 90 patients with AD will be recruited and randomized into 2 groups: intervention and control group (n = 45 per group). Random number sequences will be generated by a statistician using SAS software, and sealed envelopes will be used for randomization. The statistician who randomly assigned the participants will not be responsible for the enrollment of subjects, and another independent statistician will be responsible for uncovering at the end of the trial. In the intervention group, along with conventional drug therapy, the patients will be subjected to reminiscence therapy for 35 to 45 minutes each time, and 2 times/wk for 12 consecutive weeks. The patients in the control group will undergo conventional drug therapy. The primary outcome will be changes in Alzheimer Disease Assessment Scale-Cognitive section (ADAS-Cog) scores in patients from each group, and the secondary outcomes are the changes of Cornell scale for depression in dementia (CSDD), Neuropsychiatric Inventory score (NPI), and Barthel Index (BI) scores. Evaluation will be performed before treatment, at 4 and 12 weeks of treatment, and 12 weeks after treatment.

### 3.2 Study participants

Patients with mild-to-moderate AD from Beijing Geriatric Hospital, China.

### 3.3 Inclusion criteria

Patients presenting with all of the following criteria will be considered for study inclusion.

- Men and women aged 65 to 89 years.
- Diagnosis of probable or possible AD based on the National Institute of Neurological and Communicative Disorders and Stroke and the Alzheimer's Disease and Related Disorders Association criteria.^[25]^
- Mild to moderate stage of dementia based on the clinical dementia rating score between 1.0 and 2.0.
- Informed consent provided by the patient and/or their legal guardian.

### 3.4 Exclusion criteria

Patients with any of the following conditions will be excluded from this study.

- Diagnosis of cognitive impairment caused by vascular dementia, Lewy body dementia, frontotemporal dementia, Parkinson disease dementia, and other diseases except AD.
- Severe hearing loss.
- Serious communication barrier.
- Comorbidities of acute infection, trauma, myocardial infarction, and other emergencies.

### 3.5 Withdrawal criteria

Patients who meet either of the following criteria will be withdrawn from this study.

- Complications that affect efficacy judgments or onset of disease that may affect outcomes.
- Use of other therapies to increase the speed of recovery.

### 3.6 Recruitment

Outpatients and inpatients in the Dementia Unit will be recruited by dementia specialists. Potential participants will be able to contact the project manager via telephone and email.

### 3.7 Baseline assessment

The baseline information that will be collected is shown in Table 1 (Additional file 2).

**Table 1:**
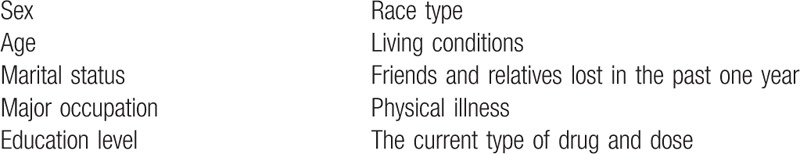
Baseline characteristics of included patients.

### 3.8 Group assignment

Patients will be randomized into control and intervention groups, and the randomization concealment will be achieved by using the sealed envelope. A code will be written on each envelope. When participants meeting the inclusion criteria are included in the trial, they will be assigned a number. The envelope with the corresponding number will be opened, and the participant will be assigned to a group indicated in the envelope. The treatment protocol for each participant will be determined by a random sequence generated by computer. Patients and the outcome evaluators will be blinded to the group assignment.

### 3.9 Interventions

Patients from both groups will receive conventional drug therapy, such as memantine, donepezil, and basic treatment for physical illnesses, diet guidance, and daily life care. The intervention group will be treated with reminiscence therapy in conjunction with conventional drug therapy the same as for the control group. For the intervention group, a grouped-based reminiscence treatment will be performed. Briefly, each 6 to 8 people from the treatment group form a subgroup, and with the guidance by a psychotherapist and 2 nurses, the group reminiscence treatment will be applied for 30 to 45 minutes per each time, 2 times/wk for 12 consecutive weeks. The specific operational procedures for the reminiscence therapy are the following: *Memories trigger* with a variety of tangible stimuli to trigger memories of patients with AD. Many of these tangible stimuli may be patient's albums, letters, and beloved items; family life supplies; drama; music; historical stories, and other stimuli, which will be obtained by the psychotherapists and nurses from the family members or insiders before the formal reminiscence treatment is started; *Memories evoked*, which also means memory extraction. Patients with AD usually have significant short-term memory impairment, whereas the long-term memory is relatively preserved, so memory extraction often point to patient pairment, whereas the long-term memory is relatively the formal reminiscence designed to gradually delve into the 12 reminiscence topics, which include diet and cooking, family relationship and early memory, old house, wedding, personal collection, working conditions and environment, songs and music, old movies, life for the first time, change and loss, celebrations, and wishes; *Presentation of the memory*—elders suffering with AD show different narrative styles. Some patients will only repeat the same story fragments, whereas others talk about a wide range of life themes and make some comments. Other patients can only give a brief description of the self-story. Therefore, medical staff need to make all the patients, as much as possible, directly involved in the interview to make the contents of the patient's story integrated, which is the most basic part of reminiscence therapy; *Memories of sharing*—repeat reading and discussion of the recalled story, to receive the benefits of reconstruction. Because AD patient's memories are often fragmented, the patient's memories will be digitally recorded, transcribed, edited, and modified so that these story fragments are reconstructed into a coordinated, coherent whole.

### 3.10 Evaluation of efficacy

The reminiscence therapy's efficacy will be evaluated by 2 attending physicians from the Dementia Unit using a specially developed assessment manual before treatment, at 4 and 12 weeks of treatment, and 12 weeks after treatment.

### 3.11 Primary outcome measure

The primary outcome is defined as the difference in ADAS-Cog scores before treatment, at 4 and 12 weeks of treatment, and 12 weeks after treatment. The ADAS-Cog, developed by Rosen et al,^[26]^ is a scale to assess cognitive function of patients with mild to moderate AD, and examines multiple cognitive domains including memory, language, praxis, and orientation. The ADAS-Cog has been well applied in trials of symptomatic treatments for mild-to-moderate AD, and its reliability and detection effectiveness have been confirmed to be acceptable by many clinical studies.^[27–29]^ Overall, it is widely used as an outcome measure in clinical trials that evaluate the cognitive changes of AD.^[30,31]^

### 3.12 Secondary outcome measures

#### 3.12.1 Cornell scale for depression in dementia

The CSDD, developed by Alexopoulos et al,^[32]^ is a valid tool for identifying depressive symptoms in patients with cognitive impairment, particularly with AD. Moreover, the validity of the CSDD has been successfully investigated and substantiated, including cross-culturally.^[33–35]^ The CSDD contains 19 questions, each of which can be given a score ranging from 0 (absent) to 2 (severe). The scale is divided into 5 subscales, including mood-related signs, behavioral disturbances, physical signs, cyclic functions, and ideational disturbance.

#### 3.12.2 Neuropsychiatric Inventory

The NPI, developed by Cummings et al,^[36,37]^ is the most widely used rating scale for psychiatric and behavior symptoms in neurologically impaired patients. The NPI examines 10 subdomains of behavioral functioning, including delusions, hallucinations, agitation/aggression, dysphoria, anxiety, euphoria, apathy, disinhibition, irritability/lability, and aberrant motor activity. Each factor will be rated for severity (0–3 points) and symptom frequency (0–4 points). The scoring index will include factor scores (frequency × severity) for a total score (0–144 points). Higher scores represent more severe neuropsychiatric symptoms. The NPI has satisfactory validity and reliability in patient settings with high scores for content validity across all items.^[36]^

#### 3.12.3 Barthel Index

This scale measures performance in activities of daily living using 10 variables, including fecal incontinence, urinary incontinence, grooming, toilet use, feeding, transfers (e.g., from chair to bed), walking, dressing, climbing stairs, and bathing. Administration of the test usually takes between 5 and 10 minutes. The scale yields a score of 0 to 100 and is interpreted as followings: ≥95 points, completely independent; 75–94 points, mildly dependent; 50–74 points, moderately dependent; 21–49 points, severely dependent; and ≤20 points, completely dependent.^[38]^

## 4 Trial timeline

**Figure 1:**
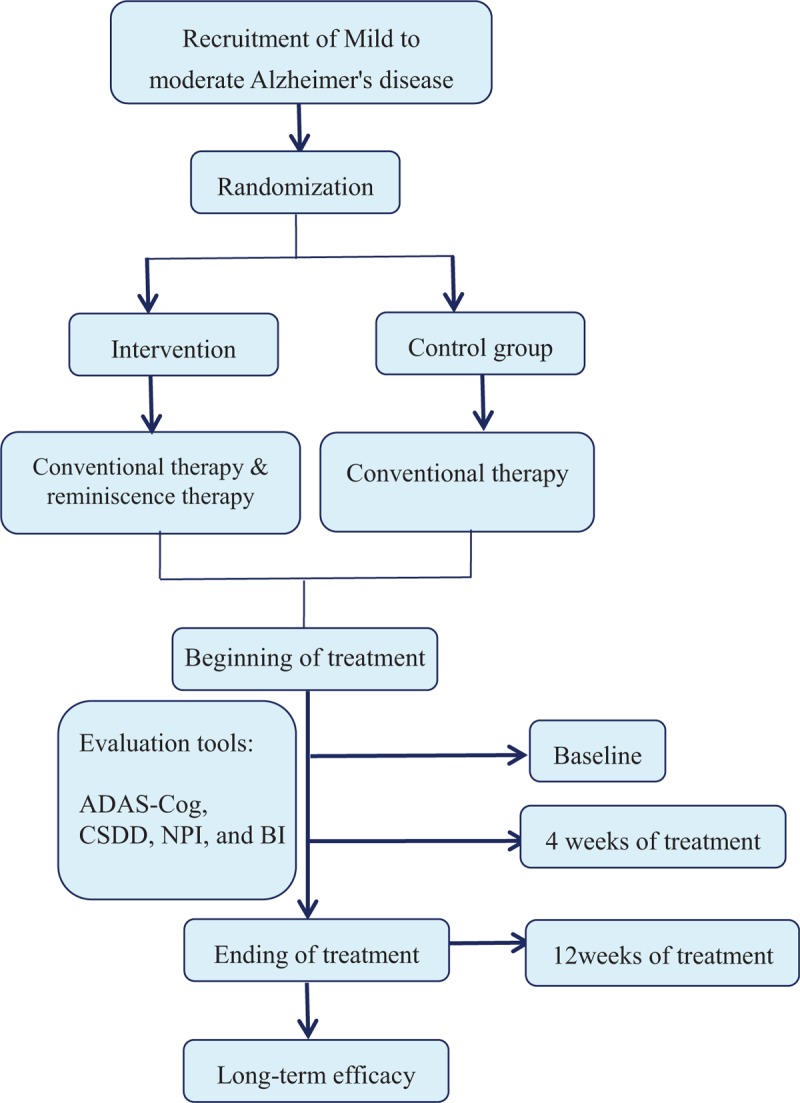
Trail protocol flowchart. ADAS-Cog = Alzheimer's Disease Assessment Scale-Cognitive section, BI = Barthel Index, CSDD = Cornell scale for depression in dementia, NPI = Neuropsychiatric Inventory score.

The schedule of outcome measurement assessments is shown in Table 2 (Additional file 3). The trial flowchart is shown in Figure 1 (Additional file 4).

**Table 2:**
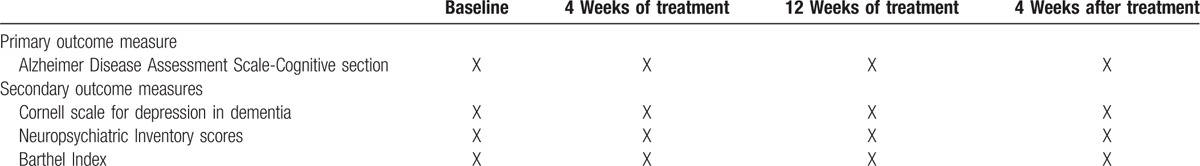
Schedule of outcome assessments.

## 5 Monitoring plan

An inspector will conduct an audit every 2 weeks. In the early stage of the trial, the inspector will obtain the clinical trial application; prepare trial protocols, clinical evaluation reports, and information consent forms; select qualified researchers; obtain institution ethics committee/institution review board approval; complete personnel training; and identify the protocols. During the trial, the inspector will focus on monitoring the test schedule, ensuring that informed consent is obtained, reviewing the case report forms, verifying the source data, assisting in creating reports regarding adverse events and serious adverse events, filing documents, and evaluating trial implementation. In the later stage of the trial, the inspector will guide the researchers in completing the necessary trial documents according to regulations and explain the responsibilities to the researchers after the trial.

### 5.1 Statistical considerations

#### 5.1.1 Sample size calculation

- We used a power of 1 – β = 0.90, with a significance level of α = 0.05. The difference in ADAS-Cog scores was used as a primary outcome between the 2 groups after treatment. Given μ_α_ = 0.05, μ_β_ = 0.10, standard deviation (σ) = 15, and tolerance error (δ) = 10, we calculated a final effective sample size for n_1_ = n_2_ of approximately 33 per group using the sample size estimation formula.
- Secondary outcome required sample size: The secondary outcomes were evaluated by CDSS, NPI, and BI, respectively. According to the previous literature data, the required samples were calculated in 32 cases, 28 cases, and 39 cases for each group, respectively.
- According to the above data, the maximum value of 39 cases for the required sample size. If we assume a patient loss rate of 15%, we will require n_1_ = n_2_ = 45 patients per group. Therefore, we decided that a sample size of 45 patients per group will participate in the trial.

#### 5.1.2 Validity evaluation

A database will be established using EpiData 3.0 software. Statistical analyses will be performed using SPSS 13.0 software (SPSS, Chicago, IL). Continuous data will be expressed as the mean ± standard deviation. The data between groups will be compared using repeated measures analysis of variance. Qualitative data will be compared using the chi-square test. The significance level will be α = 0.05, and 2-sided tests will be used.

### 5.2 Data management

Case report forms will be completed by clinical researchers, who will ensure accurate, complete, and timely data entry. Electronic data will be uploaded into EpiData 3.0 software (Epidata Association, Odense, Denmark). After checking the data, the database will be locked by the main investigator. Locked data will not be changed. The data will be statistically analyzed by statisticians. The published data will be publicly available at www.figshare.com.

### 5.3 Feasibility analysis of the present study

The reminiscence therapy approach has several key advantages. Firstly, for individuals suffering from AD, it is noninvasive, safe, with a low risk of side effects, and is generally accepted by patients. Secondly, the reminiscence therapy does not involve complex psychotherapy techniques. Therefore, it is easy to grasp by nurses or caregivers. Thus, reminiscence therapy has a great potential for clinical application.

### 5.4 Quality control

The investigator and other staff involved in the study will perform their duties, strictly follow the clinical trial protocol, and adopt standard operating procedures to ensure the implementation of the quality control and quality assurance systems. During the clinical trial, all observations and findings will be verified, and quality control will be conducted at each stage of data processing to ensure that the data are complete, accurate, authentic, and reliable. Original documents and case report forms will be examined with a focus on compliance, integrity, consistency, and severe adverse events. Severe adverse events will be reported to the ethics committee within 24 hours. Documents submitted to the ethics committee during the trial will contain test plan amendments, informed consent amendments, severe adverse event reports, and participant recruitment advertisements (if adopted).

### 5.5 Ethical requirements and informed consent

The protocols have been approved by the Ethics Committee of Beijing Geriatric Hospital of China (approval number: 2015-010). Written informed consent will be provided by the patients or their guardian after they have indicated that they fully understand the treatment plan. This article adheres to the Standard Protocol Items: Recommendations for Interventional Trials (SPIRIT) (Additional file 1).

### 5.6 Direct access to source data and files

The participants will authorize test monitors, inspectors, and the ethics committee to have access to the original medical records directly by informed consent for the verification of test procedures or test data. These authorized persons will abide by the confidentiality requirements.

### 5.7 Contents in the clinical trial report

General data (type of disease, total number of cases, and the choice of cases); clinical trial methods (settings of the control group, if necessary); statistical methods and evaluation methods; clinical evaluation criteria; clinical trial results; adverse events, side effects, and their management; analysis of clinical trial effect; clinical trial conclusion; indications, scope of application, contraindications, and precautions; problems and suggestions for improvement; clinical trial management opinion of medical institutions responsible for clinical trials.

## 6 Confidentiality

The results and protocols will be held strictly confidential by researchers. Publication of any data will be forbidden unless the sponsor authorizes it. The information provided by the sponsor, such as the protocols, design, results, data collection, case report forms, and informed consent documents will be kept confidential. No person, other than an authorized researcher, may access these documents.

### 6.1 Publication agreement of test results

Test results can be published by sponsors and authorized researchers.

## 7 Responsibilities

The ethics committee will agree on a clinical trial to ensure the rights and interests of the participants in accordance with existing data and cognitive levels. A clinical trial institution will have the ability to monitor and organize trials. The investigator will ensure that all participants are fully aware of the protocol, relevant regulations, the characteristics of the medical instruments, and their responsibilities associated with the test. The investigator will ensure that the number of participants is sufficient, the participants are in accordance with the inclusion criteria, there is sufficient time in the test period, and the trial will be compliantly and securely implemented and completed.

The sponsor is responsible for the safety and authenticity of the medical devices used in the trial and will ensure that all investigators conducting the trials adhere to the clinical trial protocol.

## 8 Results

### 8.1 Trial status

This trial has been registered in the Chinese Clinical Trial Registry (identifier: ChiCTR-INR-16009505) on October 20, 2016. Patient recruitment is ongoing.

## 9 Discussion

### 9.1 Significance of this study

Reminiscence is of potential to become a nonpharmacological therapy for the elderly who suffer from cognitive dysfunction, and patients with AD, for the following reasons. Firstly, reminiscence has been described as “the volitional or non-volitional act or process of recollecting memories of oneself in the past,”^[39]^ and is a process of recalling and re-experiencing one's life events. Moreover, this process itself will help the elderly to extract and repeat and thereby strengthening personal memories. Therefore, it is very suitable for the elderly with AD.^[40]^ Secondly, many studies reported that reminiscence therapy may help alleviate depressive symptoms in the elderly suffering from depression^[41–45]^ or cancer.^[46]^ The elderly with AD often have depressive symptoms, and the overall incidence of behavioral and psychological symptoms is reported to be as high as 70% to 90% at specific stages of AD.^[47]^ Therefore, reminiscence therapy has a positive effect not only on cognitive function, but also on emotional functions for the elderly with dementia.^[16]^ Furthermore, compared to conventional drug treatment, reminiscence treatment has no severe side effects.^[48]^ Compared to other kinds of nonpharmacological therapy, such as cognitive therapy, reminiscence treatment is easy to perform by a therapist or nurse who has been trained.^[49]^

### 9.2 Innovation of this study

Firstly, most similar previously published studies were performed in dementia patients.^[11–16,18,21,24]^ However, dementia itself is a clinical syndrome, the causes of which includes AD, vascular dementia, Lewy body dementia, frontal temporal leaf disease, and other different pathogenesis of diseases. The different underlying background diseases in patients of dementia may be the important confounding factors that affect the overall therapeutic efficacy of certain treatments for dementia. For example, the characteristics of Lewy body–related dementia is the existence of cognitive function fluctuations, regardless of treatment or not.^[50]^ Therefore, this study included patients with dementia with AD only, with the purpose of eliminating the pathological heterogeneity of dementia on the effect of intervention.

Second, most of the previous studies used the mini-mental state examination (MMSE) scale for cognitive function evaluation as the primary indicator. However, some items of MMSE may not be sensitive for the evaluation of cognitive function in dementia patients with mild AD.^[51]^ Thus, in this study we used ADAS-Cog to evaluate cognitive function, which is more widely used as a standard tool in pivotal clinical trials to detect therapeutic efficacy in cognition.^[52]^

Thirdly, in this study, CSDD, NPI, and BI are also used to evaluate the effects of reminiscence therapy on the mood, behavior, and psychiatric symptoms, as well as daily living ability of patients with AD. Because of the lack of relevant research reports in China, this study may be the first clinical trial of group reminiscence intervention for people with AD in China. Results of our study may provide additional information on the potential efficacy of reminiscence therapy on cognitive function in dementia patients with AD.

### 9.3 Evidence for contribution to future studies

The aim of this study is to evaluate the effects of reminiscence therapy on cognitive, emotional, behavioral, and psychological symptoms, and daily living ability of Chinese elderly patients with mild and moderate AD. Despite reminiscence therapy being widely used in Western countries, it is often used as an empirical application in China. The evidence-based efficacy of reminiscence therapy in patients with AD-related dementia remains to be determined. In addition, as the Chinese elderly are different from Western elderly in cultural background, values, and expression, it is necessary to verify the efficacy of reminiscence treatment in elderly Chinese patients with AD. The data and results will contribute to the efficacy of nonpharmacological interventions in AD of elderly Chinese.

## References

[1] Mucke L Neuroscience: Alzheimer's disease. Nature 2009;461:895–7. 19829367 10.1038/461895a

[2] World Health Organization (WHO). Dementia fact sheet N. 362, 2015. http://www.who.int/mediacentre/factsheets/fs362/en/

[3] Zheng S Zhang YL Zhou Z Epidemiological study of Alzheimer's disease. Chin J Gerontol 2010;30:1455–7.

[4] Cerejeira J Lagarto L Mukaetova-Ladinska EB Behavioral and psychological symptoms of dementia. Front Neurol 2012;3:73. 22586419 10.3389/fneur.2012.00073 PMC3345875

[5] Kales HC Gitlin LN Lyketsos CG Assessment and management of behavioral and psychological symptoms of dementia. BMJ 2015;350:h369. 25731881 10.1136/bmj.h369 PMC4707529

[6] Abhilash KD Faith GD Management of behavioral and psychological symptoms of dementia. Curr Geriatr Rep 2014;11:259–72.

[7] De Oliveira AM Radanovic M De Mello PC Nonpharmacological interventions to reduce behavioral and psychological symptoms of dementia: a systematic review. Biomed Res Int 2015;2015:218980. 26693477 10.1155/2015/218980 PMC4676992

[8] Bohlmeijer E Roemer M Cuijper S The effects of reminiscence on psychological well-being in older adults: a meta analysis. Aging Ment Health 2007;11:291–300. 17558580 10.1080/13607860600963547

[9] Chief, VandenBos GR. APA *Dictionary of Psychology*. 1st ed. Washington, DC: American Psychological Association; 2006.

[10] Tadaka E Kanagawa K Effects of reminiscence group in elderly people with Alzheimer disease and vascular dementia in a community setting. Geriatr Gerontol Int 2007;7:167–73.

[11] Kunz JA Targeted reminiscence interventions for older adults with dementia. J Geriatr Psychiatr 2005;35:25–49.

[12] Blake M Group reminiscence therapy for adults with dementia: a review. Br J Community Nurs 2013;18:228–33. 23752322 10.12968/bjcn.2013.18.5.228

[13] Wingbermuehle C Bryer D Berg-Weger M Baseball reminiscence league: a model for supporting persons with dementia. J Am Med Dir Assoc 2014;15:85–9. 24461238 10.1016/j.jamda.2013.11.006

[14] Gonzalez J Mayordomo T Torres M Reminiscence and dementia: a therapeutic intervention. Int Psychogeriatr 2015;27:1731–7. 25765779 10.1017/S1041610215000344

[15] Woods RT Orrell M Bruce E REMCARE: pragmatic multi-centre randomised trial of reminiscence groups for people with dementia and their family carers: effectiveness and economic analysis. PLoS One 2016;11:e0152843. 27093052 10.1371/journal.pone.0152843 PMC4836678

[16] Wang JJ Group reminiscence therapy for cognitive and effective function of demented elderly in Taiwan. Int J Geriatr Psychiatry 2007;22:1235–40. 17503545 10.1002/gps.1821

[17] Bohlmeijer E Roemer M Cuijper P The effects of reminiscence on psychological well-being in older adults: a meta analysis. Aging Ment Health 2007;11:291–300. 17558580 10.1080/13607860600963547

[18] Okumura Y Tanimukai S Asada T Effects of short-term reminiscence therapy on elderly with dementia: a comparison with everyday conversation approaches. Psychogeriatrics 2008;8:124–33.

[19] Cotelli M Manenti R Zanetti O Reminiscence therapy in dementia: a review. Maturitas 2012;72:203–5. 22607813 10.1016/j.maturitas.2012.04.008

[20] Van Bogaert P Van Grinsven R Tolson D Effects of SolCos model-based individual reminiscence on older adults with mild to moderate dementia due to Alzheimer disease: a pilot study. J Am Med Dir Assoc 2013;14:528.e9–13. 10.1016/j.jamda.2013.01.020 23583001

[21] Melendez JC Torres M Redondo R Effectiveness of follow-up reminiscence therapy on autobiographical memory in pathological ageing. Int J Psychol 2015;52:283–90. 26377350 10.1002/ijop.12217

[22] Huang HC Chen YT Chen PY Reminiscence therapy improves cognitive functions and reduces depressive symptoms in elderly people with dementia: a meta-analysis of randomized controlled trials. J Am Med Dir Assoc 2015;16:1087–94. 26341034 10.1016/j.jamda.2015.07.010

[23] Duru AG Kapucu S The effect of reminiscence therapy on cognition, depression, and activities of daily living for patients with Alzheimer disease. J Geriatr Psychiatry Neurol 2016;29:31–7. 26251112 10.1177/0891988715598233

[24] Wu LF Koo M Randomized controlled trial of a six-week spiritual reminiscence intervention on hope, life satisfaction, and spiritual well-being in elderly with mild and moderate dementia. Int J Geriatr Psychiatry 2016;31:120–7. 25965388 10.1002/gps.4300

[25] McKhanna GM Knopmanc DS Chertkowd H The diagnosis of dementia due to Alzheimer's disease: recommendations from the National Institute on Aging and the Alzheimer's Association workgroup. Alzheimers Dement 2011;7:263–9. 21514250 10.1016/j.jalz.2011.03.005 PMC3312024

[26] Rosen WG Mohs RC Davis KL A new rating scale for Alzheimer's disease. Am J Psychiatry 1984;141:1356–64. 6496779 10.1176/ajp.141.11.1356

[27] Loveman E Green C Kirby J The clinical and cost-effectiveness of donepezil, rivastigmine, galantamine and memantine for Alzheimer's disease. Health Technol Assess 2006;10:1–60. 10.3310/hta10010 16409879

[28] Li J Wu HM Zhou RL Huperzine A for Alzheimer's disease. Cochrane Database Syst Rev 2008;16 2:CD005592. 10.1002/14651858.CD005592.pub2 PMC13178729 18425924

[29] Birks J Grimley Evans J Iakovidou V Rivastigmine for Alzheimer's disease. Cochrane Database Syst Rev 2009;15 2:CD001191. 10.1002/14651858.CD001191 11034705

[30] Ihl R Ferris S Robert P Detecting treatment effects with combinations of the ADAS-cog items in patients with mild and moderate Alzheimer's disease. Int J Geriatr Psychiatry 2012;27:15–21. 21384431 10.1002/gps.2679

[31] Rozzini L Chilovi BV Conti M Conversion of amnestic mild cognitive impairment to dementia of Alzheimer type is independent to memory deterioration. Int J Geriatr Psychiatry 2007;22:1217–22. 17562522 10.1002/gps.1816

[32] Alexopoulos GS Abrams RC Young RC Cornell scale for depression in dementia. Biol Psychiatry 1988;23:271–84. 3337862 10.1016/0006-3223(88)90038-8

[33] Schreiner AS Morimoto T Factor structure of the Cornell scale for depression in dementia among Japanese poststroke patients. Int J Geriatr Psychiatry 2002;17:715–22. 12211120 10.1002/gps.684

[34] Lin JN Wang JJ Psychometric evaluation of the Chinese version of the Cornell Scale for Depression in Dementia. J Nurs Res 2008;16:202–10. 18792890 10.1097/01.jnr.0000387307.34741.39

[35] Snowdon J Rosengren D Daniel F Australia's use of the Cornell scale to screen for depression in nursing homes. Australas J Ageing 2011;30:33–6. 21395938 10.1111/j.1741-6612.2010.00450.x

[36] Cummings JL Mega M Gray K The neuropsychiatric inventory: comprehensive assessment of psychopathology in dementia. Neurology 1994;44:2308–14. 7991117 10.1212/wnl.44.12.2308

[37] Cummings JL The Neuropsychiatric Inventory: assessing psychopathology in dementia patients. Neurology 1997;48 (suppl 6):S10–6. 10.1212/wnl.48.5_suppl_6.10s 9153155

[38] Sulter G Steen C De Keyser J Use of the Barthel index and modified Rankin scale in acute stroke trials. Stroke 1999;30:1538–41. 10436097 10.1161/01.str.30.8.1538

[39] Bluck S Levine LJ Reminiscence as autobiographical memory: a catalyst for Reminiscence Theory Development. Aging Society 1998;18:185–208.

[40] Hsieh HF Wang JJ Effect of reminiscence therapy on depression in older adults: a systematic review. Int J Nurs Stud 2003;40:335–45. 12667510 10.1016/s0020-7489(02)00101-3

[41] Housden S The use of reminiscence in the prevention and treatment of depression in older people living in care home: a literature review. Groupwork 2009;19:28–45.

[42] Bohlmeijer E Kramer E Smit F The effects of integrative reminiscence on depressive symptomatology and mastery of older adults. Community Ment Health J 2009;45:476–84. 19777348 10.1007/s10597-009-9246-z

[43] Chiang KJ Chu H Chang HJ The effects of reminiscence therapy on psychological well-being, depression, and loneliness among the institutionalized aged. Int J Geriatr Psychiatry 2010;25:380–8. 19697299 10.1002/gps.2350

[44] Karimi H Dolatshahee B Momeni K Effectiveness of integrative and instrumental reminiscence therapies on depression symptoms reduction in institutionalized older adults: an empirical study. Aging Ment Health 2010;14:881–7. 20737322 10.1080/13607861003801037

[45] Wu Li-Fen Group integrative reminiscence therapy on self-esteem, life satisfaction and depressive symptoms in institutionalised older veterans. J Clin Nurs 2011;20:2195–203. 21631615 10.1111/j.1365-2702.2011.03699.x

[46] Ando M Tsuda A Moorey S Preliminary study of reminiscence therapy on depression and self-esteem in cancer patients. Psychol Rep 2006;98:339–46. 16796085 10.2466/pr0.98.2.339-346

[47] Finkel SI Costa e Silva J Cohen G Behavioral and psychological signs and symptoms of dementia: a consensus statement on current knowledge and implications for research and treatment. Int Psychogeriatr 1996;8 (suppl 3):497–500. 9154615 10.1017/s1041610297003943

[48] Jones ED Beck-Little R The use of reminiscence therapy for the treatment of depression in rural-dwelling older adults. Issues Ment Health Nurs 2002;23:279–90. 11942192 10.1080/016128402753543018

[49] Chen TJ Li HJ Li J The effects of reminiscence therapy on depressive symptoms of Chinese elderly: study protocol of a randomized controlled trial. BMC Psychiatry 2012;12:189. 23126676 10.1186/1471-244X-12-189 PMC3507717

[50] McKeith IG Dementia with Lewy bodies. Br J Psychiatry 2002;180:144–7. 11823325 10.1192/bjp.180.2.144

[51] Tombaugh TN McIntyre NJ The mini-mental state examination: a comprehensive review. J Am Geriatr Soc 1992;40:922–35. 1512391 10.1111/j.1532-5415.1992.tb01992.x

[52] Robert P Ferris S Gauthier S Review of Alzheimer's disease scales: is there a need for a new multi-domain scale for therapy evaluation in medical practice? Alzheimer Res Ther 2010;2:24. 10.1186/alzrt48 PMC2949590 20796301

